# Performance of Eco-Friendly Cement Mortars Incorporating Ceramic Molds Shells and Paraffin Wax

**DOI:** 10.3390/ma16175764

**Published:** 2023-08-23

**Authors:** Sandra Cunha, Raphael Silva, José Aguiar, Fernando Castro

**Affiliations:** 1 Centre for Territory, Environment and Construction (CTAC), Department of Civil Engineering, Campus de Azurém, University of Minho, 4800-058 Guimarães, Portugal; sandracunha@civil.uminho.pt (S.C.); raphael.rezende@live.com (R.S.); 2 Mechanical Engineering and Resource Sustainability Center (MEtRICs), Department of Mechanical Engineering, Campus de Azurém, University of Minho, 4800-058 Guimarães, Portugal; fcastro@dem.uminho.pt

**Keywords:** foundry wastes, ceramic mold shells, paraffin wax, eco-friendly mortars, freeze–thaw performance

## Abstract

The lost wax foundry industry has been rapidly expanding in recent years, generating a large amount of waste due to the fact that most of the durable goods include castings and the need for dimensional precision castings for specific purposes, such as the automotive and aeronautics sectors. The waste produced by this industry is currently being deposited in landfills because practical applications are not known and cannot be reused in a new production process, and recycling is also a challenge because of the economics of the process. Thus, the main objective of this study consists in the incorporation of the produced wastes by the lost wax casting foundry industry (ceramic molds shells and paraffin wax) as substitutes for natural aggregate in exterior coatings mortars, evaluating their behavior under normal operating conditions and against freeze–thaw actions. The obtained results revealed porosity, flexural strength, and compressive strength adequate under normal operating conditions. The freeze–thaw performance of the mortars with waste incorporation was similar to the mortars developed with natural aggregates. Thus, the potential of the ceramic mold shells and paraffinic waxes utilization in cementitious mortars for the construction sector was demonstrated.

## 1. Introduction

Presently, the world witnesses economic growth propelled by its industrial and urban advancements [1]. Consequently, an increasing number of industries are generating waste [2,3,4], which needs to be recovered to prevent landfill deposition and mitigate adverse environmental impacts. The waste production from foundry industries is escalating on a global scale [5], given that castings are an integral part of about 90% of durable goods [6]. Simultaneously, the rapid progress in global infrastructure development has resulted in a significant acceleration of construction rates [2,7]. Thus, the construction industry, a huge consumer of raw materials for the production of construction materials, namely concrete and mortars [8], needs to find sustainable and alternative solutions that allow to reduce the use of natural raw materials, namely aggregates.

In 2020, the foundry industry produced about 103,300 thousand tons of castings [9], produced by about 35,000 active foundry companies [10]. The main world producers are China, India, and the United States, representing about 50.3%, 10.9%, and 9.4% of world casting production, respectively [9]. At this time, it is estimated that around 90,000 thousand tons of foundry waste are generated worldwide annually, with only between 15% and 30% being reused, with the remainder being landfilled [11,12,13,14].

In recent times, the precision casting or lost wax casting sector has experienced remarkable growth due to its significant advantages, including high dimensional accuracy, excellent surface quality, and the ability to accommodate highly complex designs. Moreover, it offers versatile applications across various industries such as automotive, valves and accessories, domestic equipment, railway construction, naval engineering, aeronautics, electrical components, machine construction, and even civil construction [15]. As evidence of its flourishing, the lost wax casting output value in China reached USD 3.3 billion in 2021, representing a remarkable increase of 17.5% compared with 2020 [16]. During the production process, the lost wax casting industry produces mainly two different types of wastes: ceramic mold shells and paraffin wax. In the lost wax casting process, the initial step involves fabricating sacrificial pieces made of paraffin wax, which are assembled onto wax support to create a cluster. Subsequently, a ceramic mold is built by repeatedly dip-coating the cluster in a ceramic slurry to form multiple ceramic layers. Once all the ceramic layers have dried, the ceramic mold and the paraffin are subjected to an oven, removing the paraffin wax by its evaporation, obtaining in this way the first waste, the paraffin wax, after condensation. The next step consists of pouring the molten metal into the empty ceramic mold and allowing it to cool and solidify. The final step consists in breaking the ceramic mold shell and separating the individual castings, obtaining the second waste type, the ceramic mold shells [17,18]. Until now, these wastes are deposited in landfills because any practical applications are not known or economic, and during the production process, their reincorporation is not possible. Thus, the raw materials used during the lost wax casting process are used only a single time. According to data from the producers, for each ton of casting, around one ton of ceramic mold shells and about 150 kg of paraffinic wax are produced.

In the construction sector, around 48 billion tons of aggregates are consumed annually [19]. The aggregates represent about 80% of the total weight of the concrete and mortars, being its main constituent [20]. Thus, the exploitation of natural resources for making aggregates is mandatory. However, this leads to serious problems related to the high energy consumption for their extraction and also to the possibility of their depletion. In the future, natural aggregates will become less available, increasing their price and difficulty in fulfilling worldwide needs. Thus, it is essential to look for alternative materials that allow replacing the aggregate in part or totally.

Due to the large number of foundry wastes available, in recent years, many researchers have developed works searching solutions for foundry wastes, essentially foundry sand [21,22,23,24,25,26,27,28] and iron and steel foundry slags [1,17,29,30,31,32], but few studies have been conducted with wastes from the precision or lost wax casting industry. Guney et al. [23] conducted an assessment of the feasibility of reusing foundry sand waste in high-performance concrete. They replaced natural sand with foundry sand in different contents (5%, 10%, and 15%). The outcomes of their study revealed that the inclusion of foundry waste resulted in a decrease in the compression and tensile strengths, as well as in the elasticity modulus of the concrete. However, the concrete containing 10% of foundry sand exhibited similar results compared to the reference concrete. Thiruvenkitam et al. [25] also evaluated the possibility of partially replacing natural sand with foundry sand in concrete, with contents varying from 0% to 25%. The results allowed us to observe that the concrete compressive strength increases until a foundry sand content of 20%. However, the incorporation of higher foundry sand content by natural aggregate replacement leads to a slight reduction in compressive strength. The concrete with the incorporation of 20% of foundry sand presents the same compressive strength classification as the concrete with 100% of natural sand (M30). In their research, Devi et al. [30] conducted a study where they utilized steel slag as an aggregate for concrete production. They assessed the durability of the concrete incorporating steel slag under different exposure conditions. Remarkably, the concrete with steel slag demonstrated satisfactory performance, even when compared with conventional concrete, presenting a pulse velocity higher than 3.5 mm/s in various exposure conditions and a weight loss 60% lower than the traditional concrete in the acid resistance tests. Keertan et al. [20] evaluated the mechanical performance of concrete mixtures produced by replacing coarse aggregate with 40%, 45%, and 50% of steel slag, observing a better performance for a steel slag content of 50% in terms of compressive strength and denser microstructure when compared with the other mixtures.

However, even though there is already research into some foundry wastes, the application of foundry wastes from the lost wax casting process is still an area with great research needs and is underdeveloped. Taking into account the expansion of the lost wax casting industry, it is also important to develop solutions that allow the reuse of ceramic mold shells and paraffin wax waste. This research team has been making efforts in an attempt to reuse these wastes [10,12]. Thus, during these preliminary works, the focus of the work was the valorization of the ceramic mold shells and paraffin wax. The presence of these wastes leads to the development of some microcracks in the mortar microstructure, especially the presence of ceramic mold shells, due to their chemical composition. However, the incorporation of 20% of paraffin wax led to a decrease of about 17%, 27%, and 64% in the flexural strength, compression strength, and adhesion of the mortars, respectively [10]. This situation was evaluated and solved by Cunha et al. [12] in a study in which they evaluated different methods to minimize the effect of the incorporation of ceramic mold shell wastes. Three different methods were analyzed: ceramic mold shell washing, polymeric fibers incorporation, and simultaneous utilization of ceramic mold shell washing and polymeric fibers incorporation. It was possible to verify that a single washing treatment was efficient in eliminating the presence of the chemical elements that leads to the alkali-aggregate reaction.

In recent years, we have observed climate changes that arise from the significant emissions of greenhouse gases, atmospheric pollution, and excessive energy consumption [33,34]. These factors are interconnected and present cumulative effects on the climate. While the term “global warming” is commonly linked to rising temperatures, climate change also influences weather patterns worldwide, resulting in extremes of both heat and cold. Consequently, issues concerning buildings in cold regions have become critical and require comprehensive attention, particularly regarding their durability, especially the durability of exterior coating mortars [35]. Thus, several researchers have explored the freeze–thaw behavior of different composite materials. Zhao et al. [36] studied the geopolymers’ freeze–thaw behavior when subjected to 50 freeze–thaw cycles, observing a decrease in their compressive strength and destruction of the specimens after the 44th freeze–thaw cycle. Cunha et al. [37] developed a study evaluating the durability of coating mortars with the incorporation of commercial paraffin. The test results allowed us to observe that cement-based mortars with higher paraffin contents reveal a higher resistance to the freeze–thaw action and lower mass loss during the tests. Guney et al. [23] studied the behavior of cementitious materials with the incorporation of foundry sand, observing a decrease in the concrete mechanical and physical properties after freeze–thaw cycles. These studies allowed us to observe the influence of freeze–thaw actions on geopolymer concrete and cementitious materials incorporating commercial paraffin and foundry wastes. However, the evaluation of the behavior against the freeze–thaw tests of cementitious materials with the incorporation of precision foundry industry wastes (ceramic mold shells and paraffin wax) remains an underdeveloped area.

According to the previously mentioned, the main objectives of this study were the following:

- Evaluate the possibility of reusing ceramic mold shells and paraffin wax, from the lost wax foundry process, as a substitute for natural aggregate in cement mortars;
- Evaluate the physical and mechanical performance of the developed mortars in normal conditions;
- Evaluate the performance of the developed mortars subjected to freeze–thaw actions.

This is the first work carried out by the research team with the simultaneous incorporation of washed ceramic mold shells and paraffinic wax. On the other hand, the evaluation of the behavior against freeze–thaw actions in mortars with the incorporation of ceramic mold shells and paraffin wax, wastes from the precision foundry industry, or lost wax foundry industry has not yet been carried out. Thus, the novelty associated with this study is based on the following:

- Utilization of waste from the lost wax foundry industry to replace natural aggregates in mortars;
- Simultaneous incorporation of washed ceramic mold shells and paraffin wax to replace natural aggregate in mortars;
- Replacement of high contents of natural aggregate by lost wax foundry industry waste;
- Evaluation of the behavior of the developed mortars under normal operation conditions;
- Evaluation of the behavior of the developed mortars against freeze–thaw actions.

## 2. Materials and Methods

### 2.1. Foundry Wastes Preparation

The wastes were collected without any treatment, so they did not present ideal conditions to be used, being necessary to give them some methods of treatment and preparation.

#### 2.1.1. Ceramic Mold Shells

**Figure 1:**
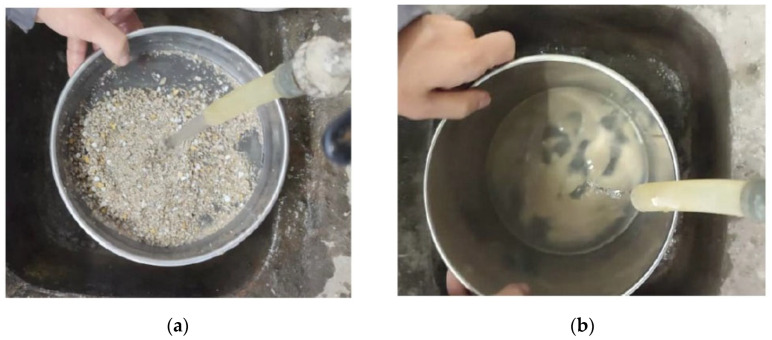
Ceramic mold shells washing process: (**a**) Washing of the particles with dimensions between 0.125 mm and 4 mm; (**b**) Washing of the particles with dimensions lower than 0.125 mm.

The ceramic mold shells were initially subjected to a process of reducing their dimensions through the use of a crusher, in which they were subjected to several grinding cycles. Subsequently, they were subjected to a sieving process in order to eliminate particles with dimensions superior to 4 mm. Finally, the ground ceramic mold shells were subjected to a washing process in order to remove the presence of some constituents that originate alkali-aggregate reactions in cementitious mixtures [12]. The washing was carried out manually using two sieves, the square mesh sieve with an opening of 0.125 mm and the sieve with an opening of 0.063 mm (Figure 1). For the ceramic mold shells with dimensions between 0.125 mm and 4 mm, the 0.125 mm sieve was used, and for ceramic mold shells with dimensions inferior to 0.125 mm, the 0.063 mm sieve was used. The washing process was carried out in accordance with the European Standard NP EN 933-1 [38].

The importance of the ceramic shell-washing process was developed in a preliminary study by the research team, and observing that this process allowed for the removal of the presence of sodium, sulfur, aluminum, potassium, magnesium, chlorides, and sulfates. Thus, the utilization of washed ceramic mold shells allows the elimination of the expansibility problems and, consequently, microcracking in the cement mortars [12]. Table 1 shows the chemical composition of the obtained crystals, resulting from a leaching procedure according to DIN EN 12457-4:2003-01 [39], obtained in a scanning electron microscope by electron dispersive spectrometry.

**Figure 2:**
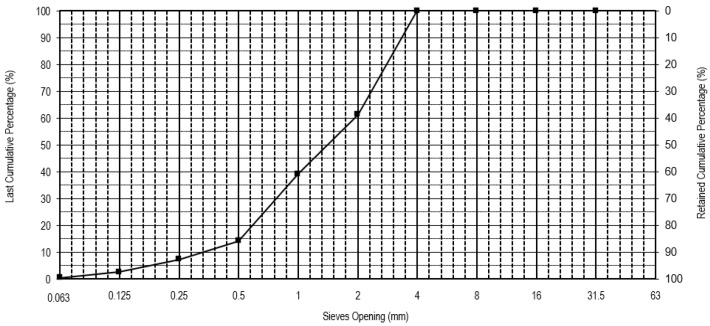
Particle size distribution of the ceramic mold shells.

Figure 2 presents the granulometry of the ceramic mold shells after grinding and washing, obtained according to the European Standard NP EN 933-1 [38]. Based on the granulometric distribution, it can be observed that a minimum dimension of 0.063 mm, a maximum dimension of 4 mm, a D10 of 0.19 mm, a D50 of 1.25 mm, and a D90 of 3.5 mm. The ceramic mold shells presented a water absorption of 6.6%.

**Figure 3:**
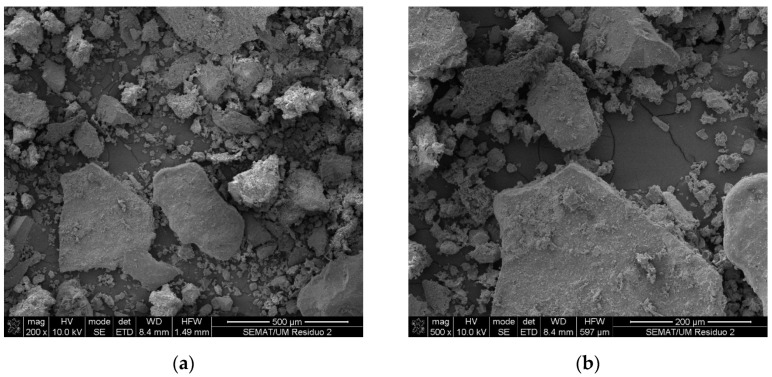
Scanning electron microscopy observations of ceramic mold shells: (**a**) magnification of 200× and (**b**) magnification of 500×.

Figure 3 presents the surface and geometry of the ceramic mold shells through scanning electron microscopy observations. It was possible to observe the existence of particles with different dimensions and angular shapes, typical of processed aggregates.

#### 2.1.2. Paraffin Wax

The paraffin wax wastes also need to be processed in order to obtain smaller particles, again using a crusher. Finally, after grinding, particles with dimensions higher than 4 mm are separated using a sieve with a 4 mm of mesh opening. These particles are rejected and inserted again in a new grinding cycle until the desired particle size is obtained.

**Figure 4:**
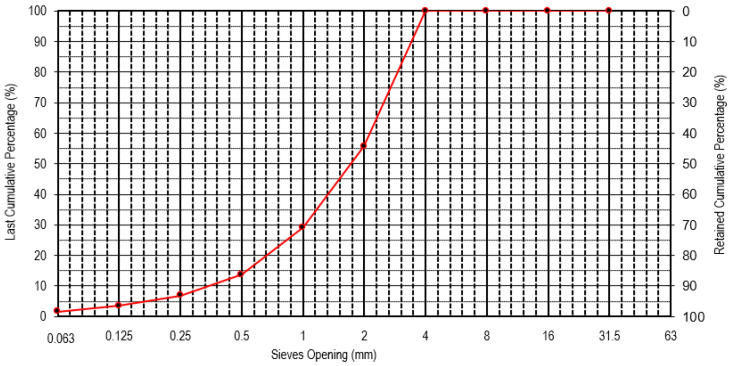
Particle size distribution of the paraffin wax [10].

**Figure 5:**
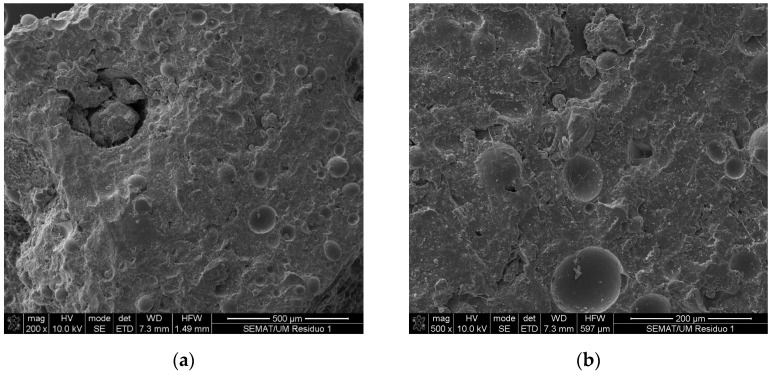
Scanning electron microscopy observations of paraffin wax waste: (**a**) magnification of 200× and (**b**) magnification of 500×.

Figure 4 presents the granulometry of the paraffin wax after grinding, obtained according to the European Standard NP EN 933-1 [38]. Figure 5 shows the paraffin wax morphology based on scanning electron microscopic observations. According to the granulometric distribution, it was possible to observe a minimum dimension of 0.063 mm, a maximum dimension of 4 mm, a D10 of 0.355 mm, a D50 of 1.8 mm, and a D90 of 3.6 mm. The paraffin wax presented a water absorption of 1.6%.

Table 2 shows the minor elements of the paraffin wax. The major elements are carbon and hydrogen. The presence of these metals in the wax may be explained by contamination of the wax during the process.

### 2.2. Materials Characterization

The materials selected for the development of mortars were based on previous work by the research team [10,12]. The Portland cement used was a CEM I 42.5R, produced by a Portuguese company (Secil, Lisbon, Portugal). The cement chemical composition is shown in Table 3, presenting a loss ignition value of 2.82%. CaO was the major chemical component, followed by SiO_2_, Al_2_O_3_, SO_3_, and Fe_2_O_3_.

**Figure 6:**
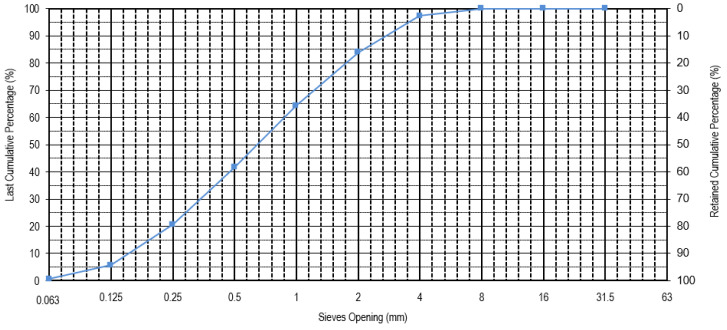
Particle size distribution of the natural sand [12].

The superplasticizer selected was based on a polyacrylate. In order to evaluate the replacement of natural aggregate, natural sand was selected with a minimum dimension of 0.063 mm and a maximum dimension of 4 mm (Figure 6). The natural sand presents a D10 of 0.16 mm, a D50 of 0.68 mm, and a D90 of 3 mm, based in their particle size distribution, obtained according to the European Standard NP EN 933-1 [38] and a water absorption of 1.19%.

Table 4 shows the density of the raw materials used to produce the different mortars.

### 2.3. Mortars and Specimens Production

Taking into account the high water absorption capacity of the used aggregates, they were initially saturated. The saturation water was mixed with the dry aggregates for 60 s, and a further 4 min were waited before continuing the production process. Then, the remaining solid materials were added to the mixture, having been mixed for 60 s.

Finally, the mixing water and the superplasticizer were added to the mixture, and the mortar was homogenized for 120 s. The different specimens were molded.

The mortar’s water absorption by capillarity, water absorption by immersion, flexural strength, and compressive strength were determined using 3 prismatic specimens for each composition and each test with dimensions of 40 mm × 40 mm × 160 mm. The behavior of mortar’s face to freeze–thaw cycles was determined using 5 specimens for each composition with dimensions of 50 mm × 50 mm × 50 mm. Regarding the microstructure observations, 2 cylindrical specimens with a diameter and height of approximately 1 cm were prepared for each mortar composition. A total of 88 specimens were produced. After the preparation, all the specimens were stored for 7 days in polyethylene bags (2 days in the mold and 5 days out of the mold). Posteriorly, the specimens were placed in a laboratory room with a controlled and constant temperature of 20 °C and humidity of 65% for 21 days.

### 2.4. Formulations

Six different mortars were developed with different materials constituting the aggregate mixture (Table 5). The cement and superplasticizer contents were fixed at 750 kg/m^3^ and 7.5 kg/m^3^, respectively. The superplasticizer content adopted was 1% of the binder weight. The volume of the aggregate and water varied according to the mortar dosage for 1 m^3^ and taking into account the maintenance of the same workability for all mortars. A reference composition with 100% aggregate constituted by natural sand (NS100) and also composition with 100% aggregate constituted by ceramic mold shells (CMS100) were developed. Four different compositions were developed with different contents of ceramic mold shells and paraffin wax (20%, 40%, 60%, and 80%).

### 2.5. Methods

The mortar’s performance was tested in the fresh state and in the hardened state. For the fresh behavior, the workability was determined according to the standard EN 1015-3 [40]. The hardened behavior was determined based on the water absorption by capillarity [41], water absorption by immersion [42], flexural strength [43], compressive strength [43], and freeze–thaw tests [44].

The microstructure observations were performed using a scanning electron microscope (EDAX-Pegasus X4M, AMETEK, Unterschleissheim, Germany).

The workability tests were performed in order to determine the mortar spreading diameter. The test procedure consists in using the spreading table standardized by the EN 1015-3 standard [40]. After producing the mortar, the mold was filled in 2 layers, compacting each one with 10 strokes. Subsequently, the excess mortar was retained from the mold, and it was vertically removed. Finally, 15 strokes were applied to the mortar. The spreading diameter was obtained through the average of two measurements perpendicular to each other of the resulting spreading mortar.

The water absorption by capillarity tests were performed following the standard EN 1015-18 [41]. The test procedure starts by drying the specimens until constant mass in an oven at 60 °C. The specimen’s lateral surfaces were coated with silicone to ensure that contact with the water happened only on the specimen’s inferior face. After drying the side waterproofing, the first contact of the specimens with a 6 mm thick sheet of water is carried out. A weighing plan was established (Table 6), starting with the dry mass (Measurement 0) and interrupted after 7 h (Measurement 8). The coefficient of water absorption by capillarity was obtained based on the slope of a linear regression obtained from the line constructed with the different measurements.

The water absorption by immersion was performed based on the Portuguese specification LNEC E 394 [42]. Initially, the samples were dried in an oven at 60 °C until constant mass (m_3_). Then, the specimens were immersed in water at a temperature of about 20 °C, at atmospheric pressure, obtaining the saturated mass (m_1_). Finally, the hydrostatic mass was determined by weighing the sample in water (m_2_). The water absorption by immersion was determined according to Equation (1) [42]:

A = ((m_1_ − m_3_)/(m_1_ − m_2_)) × 100 (1)

where

A—water absorption by immersion (%);

m_1_—saturated sample mass (g);

m_2_—hydrostatic sample mass (g);

m_3_—dry sample mass (g).

The flexural and compressive strengths of the mortars were performed in accordance with the European Standard EN 1015-11 [43]. The flexural and compressive tests were performed with load control at a speed of 50 N/s and 500 N/s, respectively. The strengths can be determined according to Equations (2) and (3) [43].

FS = 1.5 × ((F × l)/(b × d^2^)) (2)

where

FS—flexural strength (MPa);

F—maximum load (N);

l—the distance between supports (mm);

b—specimen width (mm);

d—specimen height (mm).

CS = F/A(3)

where

CS—compressive strength (MPa);

F—maximum load (N);

A—area (mm^2^).

The freeze–thaw tests were carried out based on the European specification CEN/TS 12390-9 [44]. The equipment used to carry out the tests was programmed with a temperature law in which each freeze–thaw cycle has a duration of 24 h, with a total of 56 cycles having been carried out. During each, the temperature varies between 24 °C and −18 °C. The specimens were saturated and subsequently subjected to temperature cycles. During the tests, each specimen was placed in contact with a layer of water, with the aim of the specimen absorbing the water lost by evaporation and also by ventilation of the equipment, thus ensuring that the pores of the specimens would always be saturated. The equipment was also programmed with a constant relative humidity of 90% in order to avoid weight loss due to water evaporation. The measurement of mortar degradation took into consideration the mass losses suffered in each cycle.

## 3. Results and Discussion

### 3.1. Workability

The workability tests were developed in order to maintain the spreading diameter between 195 and 205 mm. The mortar’s water/binder ratio was iteratively determined, considering the mortar’s constituent material’s density and a mortar volume of 1 m^3^.

**Figure 7:**
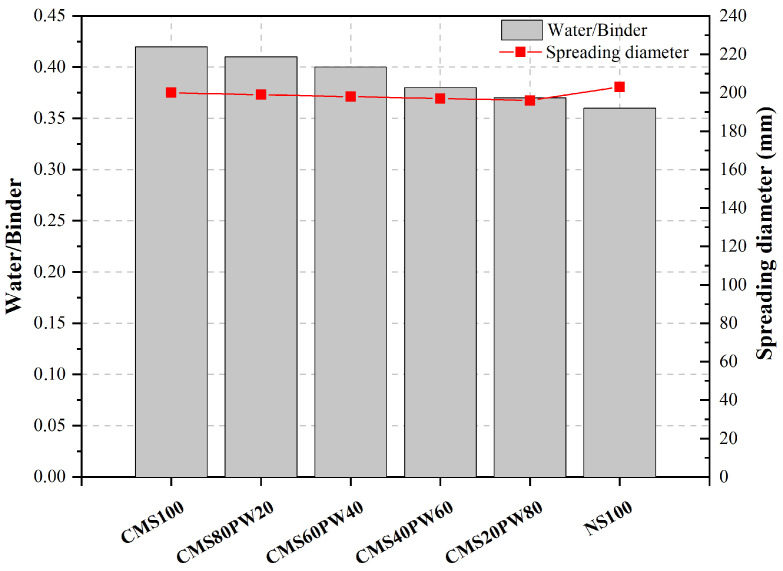
Water/binder ratio of the developed mortars.

According to Figure 7, it was possible to observe that the mortar with 100% of the natural aggregate (NS100) presents a lower water/binder ratio compared with the mortars with the incorporation of ceramic mold shells and paraffin wax. The replacement of 100% of natural sand with 100% of ceramic mold shell leads to an increase in the water/binder ratio of about 16.7%. The presence of paraffin wax in the mortar’s constituents causes an increase in the water/binder ratio higher than 2.8% compared with the NS100 mortar. This behavior can be justified by the lower water absorption capacity of the natural aggregate compared with the recycled aggregates, about 1.19%.

The mortar produced with 100% of ceramic mold shells (CMS100) shows a higher water-binder ratio, which can be justified by the more angular shape, roughness, and higher water absorption capacity of the aggregate, about 6.6%. The ceramic mold shells replacement by paraffin wax waste leads to a decrease in the water/binder ratio. The incorporation of 20% of paraffin wax causes a reduction in the water/binder ratio higher than 2.4%. This behavior can be justified by the higher average particle size of the paraffin wax particle compared with the ceramic mold shells (Figure 4) and also due to their lower water absorption capacity compared with the ceramic mold shells, about 1.6%.

### 3.2. Water Absorption by Capillarity

**Figure 8:**
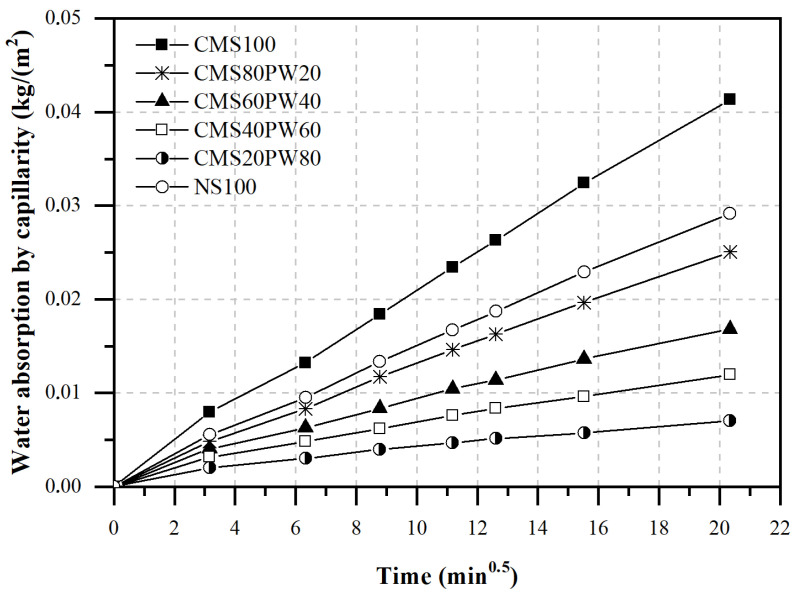
Water absorption by capillarity of the developed mortars.

**Figure 9:**
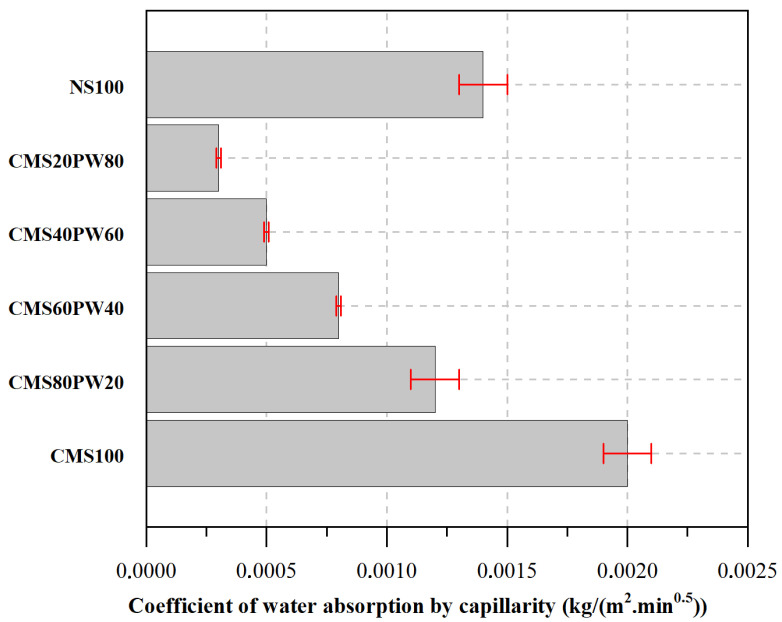
Coefficient of water absorption by capillarity of the developed mortars.

The water absorption by capillarity was evaluated based on the water absorption capacity (Figure 8) and on the coefficient of water absorption by capillarity (Figure 9).

According to Figure 8, it is possible to observe that the composition with the incorporation of 100% aggregate constituted by ceramic mold shells (CMS100) shows the greatest capacity to absorb water by capillarity during the first seven hours of contact with water. This behavior can be associated with the presence of a higher water/binder ratio of these mortars (Figure 7) and also due to the higher capacity of water absorption characteristic of the waste material. The replacement of ceramic mold shells with paraffinic wax leads to a decrease in water absorption by capillarity. The incorporation of 20% paraffinic wax results in a decrease in water absorption by capillarity of about 39%. The observed behavior can be explained by the lower ratio of water/binder present in mortars with simultaneous incorporation of the two foundry wastes (CMS80PW20, CMS60PW40, CMS40PW60, and CMS20PW80), as shown in Figure 7. The lower water absorption capacity of the paraffin wax waste is also due the higher particle dimensions of the paraffin wax compared with the ceramic mold shells average particle size. Finally, the mortars developed with the aggregate constituted by 100% of natural sand (NS100) showed a water absorption by capillarity higher than the mortars with simultaneous incorporation of ceramic mold shells and paraffin wax, even presented the lower water/binder ratio of all developed mortars. This behavior can be related to different aspects, namely the lower water absorption capacity of the natural raw material and the fact that natural sand has a smaller particle size, allowing a greater adjustment in the microstructure of the mortar.

In Figure 9, it was possible to observe the coefficient of water absorption by capillarity of the developed mortars. These results are in line with the results presented for the capillary water absorption capacity (Figure 8), with the CMS100 mortar having the highest capillary water absorption coefficient, followed by the NS100 mortar and then the mortars with simultaneous incorporation of ceramic mold shells and paraffinic wax (CMS80PW20, CMS60PW40, CMS40PW60, and CMS20PW80). The replacement of 100% of natural sand with 100% of ceramic mold shell leads to an increase in the capillary water absorption coefficient of about 42.8%. The presence of paraffin wax causes a decrease in the capillary water absorption coefficient higher than 14.3% compared with the reference mortar (NS100).

**Figure 10:**
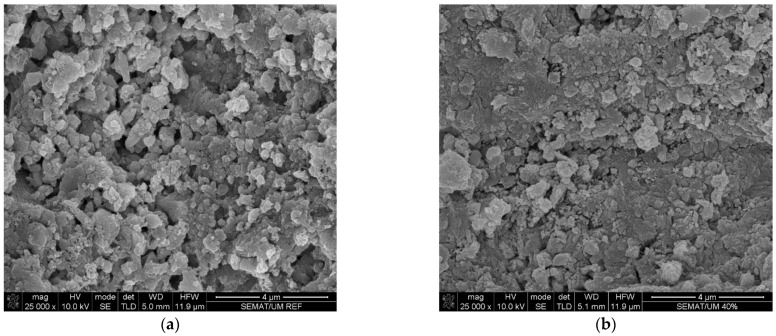
Mortar matrix scanning electron microscopy observations, magnification of 25,000×: (**a**) mortar with aggregate constituted by 100% of ceramic mold shells (CMS100); and (**b**) mortar with aggregate constituted by 60% of ceramic mold shells and 40% of paraffin wax (CMS60PW40).

According to Figure 10, it was possible to observe a more compact microstructure for the mortar with the incorporation of 40% paraffin, replacing the ceramic mold shells (CMS60PW40), proving its lower microporosity, reflected by the smaller size and quantity of pores, comparatively to the mortar with the incorporation of 100% of ceramic mold shells (CMS100). The obtained results are also in accordance with previous works developed by the research team [10,12].

### 3.3. Water Absorption by Immersion

**Figure 11:**
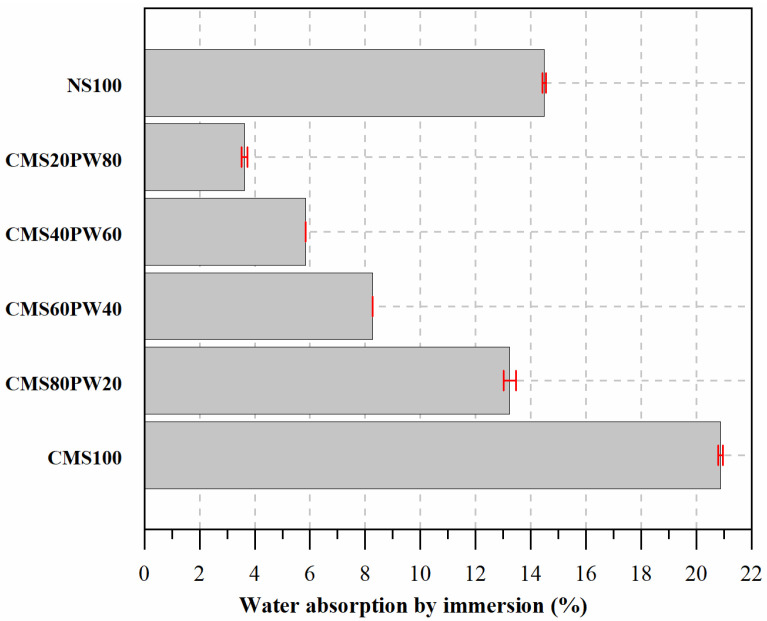
Water absorption by immersion of the developed mortars.

In Figure 11, it was possible to observe the water absorption by immersion of the developed mortars.

The mortar with incorporation of 100% of aggregate constituted by ceramic mold shells (CMS100) presents the highest water absorption by immersion. Once again, this behavior can be associated with the higher water content present in the mortars (Table 5), which leads to a more porous mortar due to the evaporation of chemically uncombined water. However, in this case, the characteristics of the recycled crushed aggregate can also contribute to greater absorption of water by immersion because this type of aggregate has a higher water absorption capacity, which, even with its initial saturation during the mortar production process, may still have some water absorption capacity. On the other hand, the ceramic mold shells have an average particle size greater than the natural sand and, also, due to their manufacturing process, a more angular shape, which provides the appearance of pores at higher dimensions. The replacement of natural sand (NS100) with ceramic mold shells (CMS100) leads to an increase in water absorption by immersion of about 44%.

The replacement of ceramic mold shells with paraffin wax translated into a decrease in water absorption by immersion. The incorporation of 20%, 40%, 60%, and 80% of paraffinic wax results in a decrease of about 37%, 60%, 72%, and 83% in water absorption by immersion, respectively. This behavior is not only associated with the presence of a smaller amount of water in these mortars (Table 5 and Figure 7) but also with the lower water absorption capacity of the paraffinic wax, around 5% inferior when compared with the water capacity of the ceramic mold shells. The obtained results are also in accordance with previous works developed by the research team [10,12].

### 3.4. Flexural and Compressive Strength

**Figure 12:**
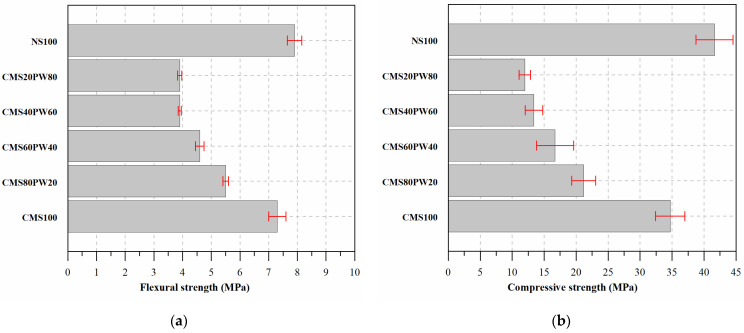
Mechanical behavior of the developed mortars: (**a**) flexural strength and (**b**) compressive strength.

Regarding the mechanical behavior (Figure 12), it was possible to observe that the mortar with the incorporation of 100% of natural sand (NS100) exhibits a higher performance, presenting high flexural and compressive strengths when compared with the mortars with the incorporation of waste materials (ceramic mold shells and paraffin wax), which can be related with the lower water/binder ratio present in these mortars.

The total replacement of the natural aggregate (NS100) by ceramic mold shells (CMS100) results in a decrease in the flexural and compressive strength of about 8% and 17%, respectively. Once again, this behavior can be justified by the higher water content present in the mortar’s composition (Table 5) and consequent higher water absorption by capillarity and immersion (Figure 8, Figure 9 and Figure 11), resulting in a greater mortar porosity.

The paraffin wax incorporation causes a decrease in flexural and compressive strength. The addition of 20% of paraffin leads to a decrease in the flexural and compressive strength of more than 24% and 39%, respectively. In this case, the observed behavior is associated with the lower adhesion of the paraffin wax to the cementitious matrix and the lower resistance of the paraffin wax particles compared with the ceramic mold shell particles [10,12]. Some studies report that the incorporation of free and pure paraffin in cementitious mixtures also indicates a delay in the cement hydration process, which may also explain the lower mechanical performance observed for mortars with higher content of paraffinic wax incorporation [45]. Even having verified a decrease in the flexural and compressive strengths of the developed mortars, it is important to note that its application in the construction industry was not compromised. According to European Standardization NP EN 998-1 [46], which provides the classification of coating mortars according to their compressive strength, the developed mortars presented the maximum classification provided, CSIV, which is obtained with a compressive strength equal to or higher than 6 MPa.

### 3.5. Freeze–Thaw Tests

The tests started in cycle 0, in which the specimens presented their mass unaltered, 100% of the mass.

**Figure 13:**
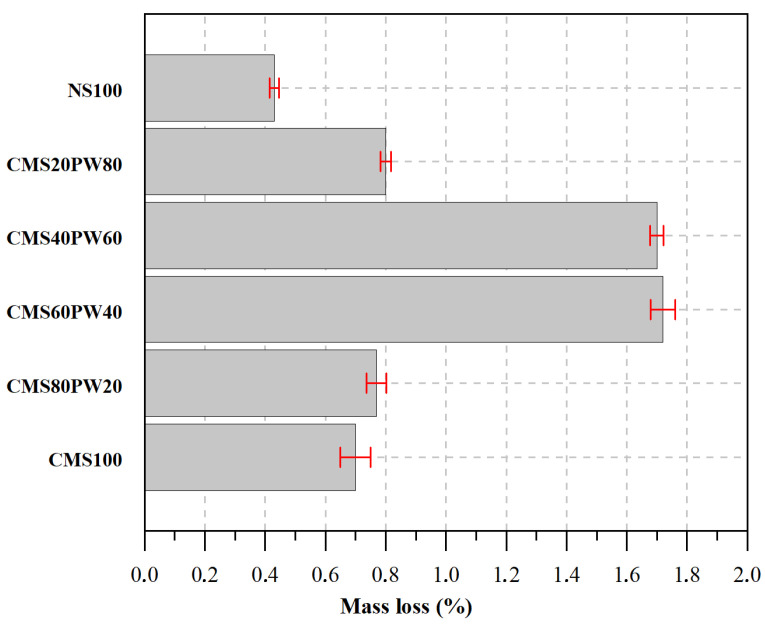
Total mass loss during the freeze–thaw tests of the developed mortars.

In Figure 13, it was possible to observe that the mortars constituted by 100% of natural sand (NS100) showed lower mass loss and faced freeze–thaw cycles due to the higher compressive strength (Figure 12). The mortar with the replacement of natural sand by 100% of ceramic mold shells (CMS100) exhibited a mass loss slightly higher than the reference mortar, which is related to its higher porosity (Figure 9 and Figure 11) and lower compressive strength (Figure 12). The higher porosity of the mortars will favor a higher water storage capacity, which, consequently, with the volume variation relative to the freezing and thawing cycles, will cause internal tensions in the microstructure of the mortar, originating the appearance of microcracks, which allow the specimen’s degradation, resulting in a higher mass loss.

The incorporation of paraffin wax in higher contents leads to a higher mass loss during the freeze–thaw tests, which is directly connected to the lower mechanical performance of the mortars due to the lower resistance of the paraffin wax compared with the ceramic mold shells (Figure 13), even presenting lower porosity. However, it is important to observe that all developed mortars exhibit an appropriate behavior to freeze–thaw action because, for all developed mortars, the mass loss is lower than 1.7%, and even the developed mortars present high levels of wastes incorporation (ceramic mold shells and paraffin wax). Another study evaluated the incorporation of paraffin directly into cementitious mixtures against freeze–thaw actions, revealing higher mass losses [37], which is justified by the lower mechanical performance of the developed mortars compared with the mechanical performance of the mortars presented in this study. The embrittlement of cementitious materials with the incorporation of wastes from the foundry industry, in particular, foundry sands subjected to freeze–thaw cycles, was also reported in another study [23].

## 4. Conclusions

This study allowed the evaluation of the influence of the incorporation of ceramic mold shells and paraffinic wax wastes in cement mortars, replacing the natural aggregate and evaluating their physical, mechanical, and freeze–thaw behavior.

Taking into account the high amount of precision foundry wastes currently generated around the world and the continuously growing prospection of this industry, it will be expected that this study has enormous applicability worldwide due to the need to reuse these wastes, but also to the need of the construction industry in reduce the natural raw materials consumption.

The most relevant conclusions achieved were the following:

- The water content in the mortars increases with the total replacement of natural sand by ceramic mold shells. However, the replacement of ceramic mold shells with paraffinic wax leads to a decrease in the water/binder ratio. These behaviors were directly connected with the particle size and water absorption capacity of the different aggregates.
- The water absorption by capillarity and immersion of the mortars with simultaneous incorporation of ceramic mold shells and paraffinic wax were lower compared with the mortar with 100% of natural sand and 100% of ceramic mold shells due to the lower ratio of water/binder of these mortars and lower water capacity absorption of the paraffinic wax. The higher mortar porosity was presented by the mortars with 100% of aggregate constituted by ceramic mold shells due to the higher water/binder ratio and water capacity absorption of the ceramic mold shells.
- The higher mechanical performance was presented by the mortar with 100% aggregate constituted by natural sand. The presence of ceramic mold shells and paraffinic wax leads to a decrease in the flexural and compressive strengths due to the lower adhesion of the paraffin wax to the cementitious matrix and the delay in the cement hydration process.
- The freeze–thaw behavior of the developed mortars was very satisfactory because all the mortars presented mass losses very reduced and close to the mortar with 100% of natural aggregate.
- The replacement of natural sand by foundry waste (peels from ceramic molds and paraffinic wax) in the compositions CMS100, CMS80PW20, CMS60PW40, CMS40PW60, and CMS20PW80 will lead to a 100% saving in the aggregate acquisition cost for these mortars.

Considering the mechanical performance (flexural and compressive behavior) and the performance against freeze–thaw actions, it was possible to observe that the mortars with the incorporation of 100% of ceramic mold shells (CMS100) and 80% of ceramic mold shells and 20% of paraffin wax (CMS80PW20) showed the more interesting behavior.

It can be concluded that the reutilization of these wastes (paraffin waxes and ceramic mold shells) as a replacement for natural aggregate in mortars can be seen as a viable solution for decreasing the consumption of natural raw materials. On the other hand, the reuse of these wastes, which so far do not have any type of application and cannot be reused during a new foundry production process, will result in a substantial reduction in their deposit in landfills.

## Figures and Tables

**Table 1:** Chemical composition of obtained crystals from the water resulting from the ceramic mold shells washing [10].

Element	wt (%)
Oxygen (O)	59.4
Silicon (Si)	15.9
Calcium (Ca)	11.8
Chlorine (Cl)	3.9
Sodium (Na)	3.3
Sulfur (S)	2.0
Potassium (K)	1.8
Phosphorus (P)	1.1
Magnesium (Mg)	0.4
Aluminum (Al)	0.4

**Table 2:** Chemical composition of paraffin wax (minor elements) [10].

Element	Weight (%)
Sodium (Na)	0.4
Aluminum (Al)	0.2
Calcium (Ca)	0.9

**Table 3:** Chemical composition of cement CEM I 42.5 R.

Chemical Component	wt (%)
Loss on ignition	2.82
Insoluble residue	1.70
Silicon oxide (SiO_2_)	19.65
Aluminum oxide (Al_2_O_3_)	4.28
Iron oxide (Fe_2_O_3_)	3.35
Calcium oxide (CaO)	61.35
Magnesium oxide (MgO)	1.70
Sulfates (SO_3_)	3.36
Potassium oxide (K_2_O)	0.89
Sodium oxide (Na_2_O)	0.19
Chlorides (Cl^−^)	0.04

**Table 4:** Materials densities (kg/m^3^).

Material	Density
CEM I 42.5R	3049
Superplasticizer	1041
Ceramic mold shells	2630
Paraffin wax	1013
Natural sand	2569

**Table 5:** Mortars formulation (kg/m^3^).

Composition	Cement	Ceramic Mold Shells	Paraffin Wax	Natural Sand	Superplasticizer	Water
NS100	750	0	0	1225	7.5	273.3
CMS100	750	1136	0	0	7.5	315.2
CMS80PW20	750	699	175	0	7.5	305
CMS60PW40	750	427	284	0	7.5	303
CMS40PW60	750	234	351	0	7.5	285
CMS20PW80	750	103	412	0	7.5	280

**Table 6:** Weighing plan of the water absorption by capillarity tests.

Measurement	Time (Hours)	Time (Minutes)
1	0.0	0
2	0.2	10
3	0.7	40
4	1.3	77
5	2.1	125
6	2.7	159
7	4.0	241
8	7.0	420

## Data Availability

Not applicable.
